# Correlation between academic hardiness and subjective well-being among teenagers: the chain mediating role of academic passion and academic self-efficacy

**DOI:** 10.3389/fpsyg.2025.1517977

**Published:** 2025-03-13

**Authors:** Lihua Zhou, Mingdan Tang, Xuejuan Du, Jian Chen

**Affiliations:** ^1^College of Education Science, Hengyang Normal University, Hengyang, Hunan, China; ^2^No. 1 Middle School of Zhong Provinece, Chongqing, China; ^3^School of Safety and Management, Hunan Institute of Technology, Hengyang, Hunan, China

**Keywords:** academic hardiness, academic passion, harmonious passion, obsessive passion, academic self-efficacy, subjective well-being, teenagers

## Abstract

Enhancing the positive qualities of adolescents and their capacity to actively attain well-being is a crucial objective in education. The correlation among academic hardiness, academic passion, academic self-efficacy and subjective well-being was explored by an investigation which was conducted among 805 junior high school students (ages 12–15) using the Academic Hardiness Scale, Academic Passion Scale, Academic Self-efficacy Scale and Subjective Well-being Index Scale in this study. The findings indicated that academic hardiness is the variable most closely related to subjective well-being; academic self-efficacy plays a significant mediating role between academic hardiness and subjective well-being; academic hardiness is positively associated with subjective well-being through the chain mediation pathway from academic harmonious passion or obsessive passion to academic self-efficacy. These results suggest that cultivating academic hardiness in teenagers is positively significant for improving their positive academic emotional experience, academic self-efficacy and overall subjective well-being.

## Introduction

The subjective well-being of adolescents is a significant influencing factor of their current (Teodora et al., 2021; Yu, 2022) and future physical and mental health as well as their adult socioeconomic status (Kansky et al., 2016; Richards and Huppert, 2011). The subjective well-being of adolescents is attracting increasing attention from society as a whole (Alsarrani et al., 2022). However, some studies have reported a rapid decline in subjective well-being among individuals in middle school (Marquez and Long, 2020; Thapar et al., 2021). Academic development has always been a developmental task of great concern for adolescents (Li and Zhang, 2014). Previous studies have suggested that the high academic achievement of adolescents contributes to the satisfaction of their autonomous needs and relationship needs, thereby promoting their subjective well-being (Huang et al., 2024; Ng et al., 2015). The general personality, emotion, and cognition factors that best predict subjective well-being have been studied through multiple lenses. However, it remains unclear how these factors of academic development correlate with subjective well-being, especially among teenagers, who are in the early stage of puberty and the transition period from elementary school to middle school. This study focuses on the relationships between academic personality, emotions, self-cognition, and subjective well-being. The results of this study have positive implications for the promotion of teenagers’ long-term academic development as well as subsequent educational practices.

## Academic hardiness and subjective well-being

Benishek and Lopez (2001) proposed the concept of academic hardiness, a sub-concept of personality hardiness in academic settings, based on the hardiness theory (Kobasa et al., 1982; Kobasa et al., 1993) and motivation theory (Dweck and Leggett, 1988) to illustrate students’ capacity to rebound from academic setbacks. Commitment, Control, and Challenge are three dimensions (3C) that describe academic hardiness (Benishek et al., 2005). Commitment reflects the extent to which individuals are willing to exert effort to improve their academic achievement when faced with academic challenges and pressure. Challenge is used to evaluate students’ purposeful engagement in difficult academic tasks and their perception of failures in academic situations as preparation for future success. Control refers to an individual’s belief in their academic abilities, specifically the belief that they can cope with academic pressure and challenges to achieve their expected goals. Therefore, academic hardiness reflects the differences in individuals’ cognition, emotions, and abilities when facing academic challenges.

Research on academic hardiness has shown that it could distinguish between students who pursue academic success and those who avoid academic difficulties, as well as the differences in their responses to academic setbacks (Creed et al., 2013). Students with low academic hardiness tend to exhibit more academic procrastination and experience higher levels of stress in their studies (Sepiadou and Metallidou, 2022). In contrast, those with high academic hardiness are more motivated to learn (Abdollahi and Noltemeyer, 2018), better equipped to handle academic stress (Kamtsios and Karagiannopoulou, 2015; Abdollahi et al., 2020), and more likely to achieve great academic success (Abdollahi and Noltemeyer, 2018; Kamtsios and Karagiannopoulou, 2015).

Personality traits are considered important internal factors that affect a person’s subjective well-being (Chuning et al., 2024). Personality hardiness helps individuals focus on the positive meaning of stressful events and take proactive actions to deal with challenges, which also serves as a protective factor for subjective well-being (Chuning et al., 2024). Wardani (2020) found that academic hardiness directly influences the psychological well-being of students. Based on a literature review of the positive relationship among academic hardiness, academic development and subject well-being, this study proposes Hypothesis 1: The academic hardiness of teenagers is positively correlated with their subjective well-being.

## The potential mediating effect of academic passion

Passion is a state of heightened physiological arousal that activates an individual’s behavior, driving them to invest time and energy into a particular activity. Academic passion signifies an individual’s affection for and value placed on learning, and their willingness to dedicate time and effort in their studies (Kamtsios and Karagiannopoulou, 2015). The dualistic model of passion (Kamtsios and Karagiannopoulou, 2015) categorizes academic passion into harmonious passion and obsessive passion based on internal and external academic behavioral motivation characteristics. If an individual engages in learning activities out of internal interest and personal values, they are more likely to experience harmonious passion. Conversely, if an individual participates in learning activities due to external pressure or others’ expectations, they are more likely to experience obsessive passion. Research has found that harmonious passion is positively correlated with students’ vitality and physical and mental health in learning (Stoeber et al., 2011). In a state of harmonious passion, individuals consciously internalize these positive experiences into their self-concept and behavior, perceiving that their actions are also under their control (Lopez and Vallerand, 2020; Ruis-Alfonso and Leon, 2016). Obsessive passions result from internalized external control, where individuals may be engaged in activities that conflict with their subjective desires or are beyond their control (Lopez and Vallerand, 2020; Kamtsios, 2022; Vallerand, 2012). Obsessive passion may lead to some maladaptive outcomes, such as negative emotional experiences (Kamtsios, 2022) or certain experiences being internalized in an uncontrolled manner (Ruis-Alfonso and Leon, 2016).

Personality traits are important influencing factors of individual passion experiences (Daple et al., 2019; Kamtsios and Karagiannopoulou, 2020; Karagiannopoulou et al., 2018). Individuals with autonomous orientation personalities are more likely to experience harmonious passion (Balon et al., 2013; Daple et al., 2019). On the contrary, individuals who are circumstance-oriented or circumstance-controlled are more likely to feel that their behavior is being manipulated by external forces, which can lead to obsessive passion (Balon et al., 2013). Individuals with high levels of academic hardiness may have a higher internal motivation to engage in academic activities in order to improve academic achievement, and are also more likely to participate in academic activities under external pressure. Kamtsios (2022) found that commitment, control, and challenge are significantly positively correlated with harmonious passion, commitment is significantly positively correlated with obsessive passion, while control and challenge are not significantly correlated with obsessive passion. Research indicates that those with elevated academic hardiness may cultivate a compulsive learning attitude under intense competitive pressure or high expectations, and are susceptible to experiencing obsessive passion (Benishek et al., 2005). This passion drives them to over engage in learning, even if it may require sacrificing other activities (Benishek and Lopez, 2001). The research results of Kamtsios (2022) also indicate that academic hardiness can indirectly affect subjective well-being through academic passion. This study proposes Hypothesis 2: Academic passion has a mediating role in the association between academic hardiness and subjective well-being in teenagers. Academic hardiness exhibits a positive correlation with harmonious passion, which in turn is positively connected with subjective well-being; academic hardiness also shows a positive correlation with obsessive passion, while obsessive passion is adversely correlated with subjective well-being.

## The potential mediating effect of academic self-efficacy

Academic self-efficacy is an individual’s belief in their capacity to manage and implement the necessary actions to achieve particular achievements (Liang, 2000). Individuals with high levels of academic hardiness are able to cognitively accept challenging tasks (challenges) and make efforts toward these tasks (commitments), and also believe that they can achieve their goals through their own efforts (control). Logically, these three aspects of academic hardiness may have a positive correlation with academic self-efficacy. Some researchers have found positive associations between hardiness and academic self-efficacy in middle school students (Wong et al., 2019), college students (Maddi et al., 2011), and graduate students (Cheng et al., 2019). Kuo et al. (2021) found a positive association between individual academic hardiness and self-efficacy in online learning environments. Cognitive behavioral theory (Bandura and Bandura, 1986) posits that when individuals believe they have the ability to perform a task well, they are more willing to invest time and energy in the task and are more likely to succeed. Academic self-efficacy can increase students’ academic engagement and positively correlate with individual academic achievement (Liu, 2022) and life satisfaction (Liu, 2022; Zhou et al., 2023). This study proposes Hypothesis 3: Academic hardiness is positively correlated with their subjective well-being through a positive association with academic self-efficacy among middle school students.

## The potential chain-mediating role of academic passion and academic self-efficacy

Individuals with academic hardiness persist in learning behaviors even when encountering difficulties in learning, which may be autonomously internalized learning activities related to academic harmonious passion or activities driven by the external requirements related to academic obsessive passion. Academic passion has a priming effect on academic behavior (Vallerand, 2012). The previous analysis indicates that both harmonious passion and obsessive passion can elevate students’ academic engagement, thereby improving their academic academic self-efficacy and further enhancing their achievement. The self-determination theory (Deci and Ryan, 2000) suggests that if an individual feels capable of autonomously choosing and controlling their behavior, achieving their chosen goals, and establishing meaningful connections with others, they are more likely to experience a harmonious relationship with their environment. This alignment is essential for both health and well-being Middle school students with high levels of academic hardiness are more likely to achieve the three basic needs proposed in self-determination theory. This study proposes Hypothesis 4: Academic passion and academic self-efficacy have a chain mediating role in the relationship between academic hardiness and subjective well-being. Specifically, academic hardiness is expected to correlate with subjective well-being through a sequential mediation pathway from academic passion to academic self-efficacy.

## The present study

Based on the above theoretical and empirical research analysis, this study suggests that there may be the following relationships between the variables of academic hardiness, academic passion, academic self-efficacy, and subjective well-being among teenagers: academic hardiness is positively correlated with subjective well-being among teenagers; academic passion (harmonious or obsessive) plays a mediating role in the relationship between academic hardiness and subjective well-being; academic self-efficacy plays a mediating role in the relationship between academic hardiness and subjective well-being; academic passion (harmonious or obsessive) and self-efficacy play a chain mediating role between academic hardiness and subjective well-being. According to the literature review above, harmonious passion is positively associated with subjective well-being, while obsessive passion is negatively associated with subjective well-being. Therefore, this study uses two chain mediation models to examine the links among the variables mentioned above.

## Materials and methods

### Participants

A total of 920 questionnaires were collected, and 805 valid questionnaires of participants aged from 12 to 15 years old (M*_*age*_* = 13.25, SD = 0.82) were obtained for an effective response rate of 81.31% by convenience sampling to collect samples from three junior high schools in Hengyang City, Zhuzhou City, Hunan Province, and Luoyang City, Henan Province. Among them, there were 524 grade 7*^th^*, 281 grade 8*^th^*; 419 male students (52.05%) and 386 female students (47.95%); 403 rural students (50.06%) and 399 urban students (49.57%), of which three students did not report their place of origin. Informed consent was obtained from the parents and responsible teachers of the participants prior to the survey.

## Instruments

### Academic hardiness scale

This study translated the *Academic Resilience Scale* developed by Benishek and Lopez (2001) and conducted a cultural adaptability test to evaluate the academic hardiness of subjects. Three psychology graduate students and two psychology associate professors used back translation procedures to form a Chinese version. They made cultural adjustments to some items based on Chinese expression habits, ultimately forming the formal scale used in this study. Project analysis and exploratory factor analysis were conducted on survey data from 265 teenagers (133 males, aged 12∼15). The *KMO* value of the scale is 0.83, and the Bartlett test of sphericity value is 1087.19 (*df* = 136, and *p* < 0.001), indicating that the scale was suitable for factor analysis. Conduct confirmatory factor analysis and criterion validity analysis were conducted on survey data from an additional 500 participants (245 males, 355 females, aged 12∼15). The model fit indices of the scale were as follows: χ^2^/df = 2.45, RMSEA = 0.05, GFI = 0.94, AGFI = 0.92, CFI = 0.90, NFI = 0.85, IFI = 0.90, TLI = 0.89. These results indicate that the scale has good construct validity. The final Chinese version of the academic hardiness scale includes 13 items and three dimensions (commitment, control, and challenge). All items are scored on a 4-point scale, with higher total scores indicating higher levels of individual academic hardiness. In this study, the Cronbach’s alpha coefficients of the total scale, commitment, control, and challenge subscale are 0.79, 0.74, 0.68, and 0.61, respectively.

### Academic passion scale

The Chinese version of the *Academic Passion Scale* (Vallerand and Houlfort, 2003; Liang and Tian, 2018) was used in this study. This scale consists of 17 items, divided into two sections. The first section consists of five items designed to evaluate whether students possess academic passion; the second section encompasses two dimensions: harmonious passion and obsessive passion, each containing six items. All items are scored on a 7-point scale, with “1” representing “completely disagree” and “7” representing “completely agree.” Higher total scores indicate higher levels of passion in the respective dimensions. The scale has demonstrated good reliability among Chinese students (Li et al., 2021). In this study, the Cronbach’s alpha coefficients of the total scale, harmonious passion, and obsessive passion subscale are 0.90, 0.87, and 0.71, respectively.

### Academic self-efficacy scale

The Chinese *Academic Self-efficacy Scale* was developed by Liang (2000). Participants rated 22 items on 5-point Likert scale, with “1” representing “strongly disagree” and “5” representing “strongly agree.” The higher the score of the subjects, the higher their level of academic self-efficacy. The Chinese version of the scale has been demonstrated previously to be a reliable and valid measure in Chinese culture (Chen et al., 2021). In this study, the Cronbach’s alpha of this scale was 0.87.

### Subjective well-being index scale

The *Subjective Well-being Index Scale* was developed by Campbell (1976) and revised by Li and Zhao (2000). The scale comprises of nine items, with eight items assessing the life satisfaction index of the participants and one item testing the overall emotional index of the participants. Higher scores on the scale indicate a higher subjective well-being index for the individual. This scale has been demonstrated previously to have satisfactory internal consistency (Wang et al., 2017). The Cronbach’s alpha coefficients of the scale in this study is 0.93.

### Data analysis

Statistical Product and Service Solutions (SPSS) version 22.0 was used to conduct a common method bias test, compute descriptive statistics for each study variable, and conduct correlation analyses. The hypothetical chain mediating role was analyzed by SPSS PROCESS (Hayes, 2013) macro (Model 6). Bootstrap procedures were conducted to test the models. Five thousand bootstrap resamples were set to calculate the 95% confidence intervals of the indirect effects in all statistical analyses. All continuous variables were standardized prior to analyses.

## Results

### Preliminary analyses

This study used a self-rated questionnaire to assess potential issues with common method bias. Harman’s one-factor test was conducted to test for common method bias. The results show 12 factors with an eigenvalue of greater than 1. The explanation rate of the first common factor was 19.96%, which was significantly lower than 40% threshold. Therefore, there was no serious common method bias in this study (Tang and Wen, 2020).

Table 1 presents the descriptive statistics and correlation analysis results of each study variable. Academic hardiness was significantly positively associated with academic passion, academic self-efficacy and subjective well-being. Academic self-efficacy was significantly positively associated with academic passion and subjective well-being. Subjective well-being was significantly positively related to harmonious passion but not significantly positively related to obsessive passion.

**TABLE 1:** Descriptive statistics and correlations of study variables.

	M	SD	1	2	3	4	5
1 Academic hardiness	46.67	5.66	1	–	–	–	–
2 Harmonious passion	28.01	7.78	0.48**	1	–	–	–
3 Obsessive passion	21.69	6.78	0.19**	0.48**	1	–	–
4 Academic self-efficacy	69.44	12.47	0.61**	0.57**	0.22**	1	–
5 Subjective well-being	40.91	11.89	0.34**	0.24**	0.01	0.30**	1

*N* = 805, **p < 0.01.

### Test of mediating effect

This study used the SPSS PROCESS macro (Model 6) to further test the multiple mediating effects among five variables. Academic hardiness was designated as the independent variable; Subjective well-being served as the dependent variable; academic passion (harmonious passion and obsessive passion) and academic self-efficacy were used as mediating variables; gender and age were included as covariates.

The results of regression analysis showed (see Table 2) that academic hardiness significantly predicted subjective well-being (β = 0.32, *p* < 0.001); academic hardiness significantly positively predicted both academic passion (harmonious passion β = 0.47, *p* < 0.001; obsessive passion β = 0.20, *p* < 0.001) and academic self-efficacy (β = 0.45 and 0.59, *p* < 0.001); academic passion significantly positively predicted academic self-efficacy (harmonious passion β = 0.36, *p* < 0.001; obsessive passion β = 0.11, *p* < 0.001) and subjective well-being (harmonious passion β = 0.05, *p* = 0.203; obsessive passion β = −0.07, *p* = 0.045); academic self-efficacy significantly negatively predicted subjective well-being (β = 0.12 and 0.16, *p* < 0.01 and *p* < 0.001).

**TABLE 2:** Regression analysis of the relationship between variables in chain mediation model.

Regression equation		Overall fit index			Significance of regression coefficients	
**Result variables**	**Predictive variables**	***R***	***R* ^2^**	***F***	**β**	***t***
Subjective well-being	Academic hardiness	0.35	0.13	38.57***	0.32	9.75***
Academic passion	Academic hardiness	0.48 (0.20)	0.23 (0.04)	78.19*** (10.92***)	0.47 (0.20)	15.00*** (5.68***)
Academic self-efficacy	Academic hardiness	0.69 (0.63)	0.48 (0.40)	185.58*** (130.57***)	0.45 (0.59)	15.35*** (20.92***)
	Academic passion	–	–	–	0.36 (0.11)	12.27*** (3.84***)
Subjective well-being	Academic hardiness	0.37 (0.38)	0.14 (0.14)	26.16*** (26.72***)	0.23 (0.24)	5.31*** (5.78***)
	Academic passion	–	–	–	0.05 (−0.07)	1.27 (−2.01*)
	Academic self-efficacy	–	–	–	0.12 (0.16)	2.65** (3.70***)

The data in parentheses represents obsessive passion. *p < 0.05, **p < 0.01, ***p < 0.001.

**FIGURE 1:**
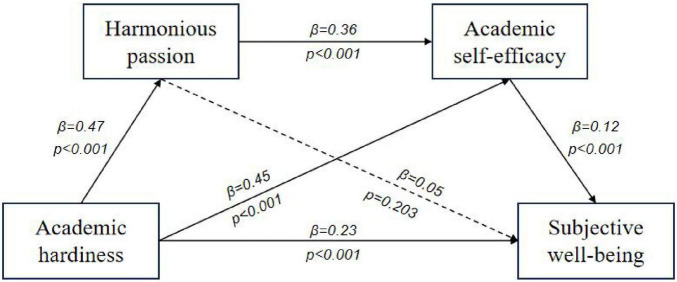
Model diagram of the effect of harmonious passion.

**FIGURE 2:**
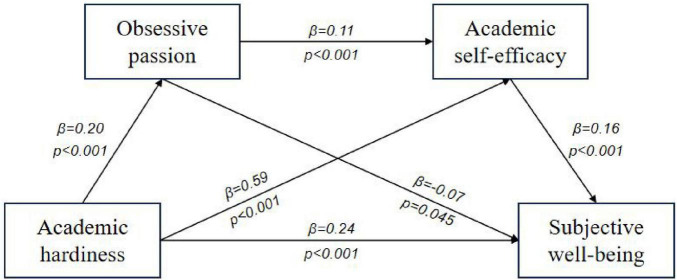
Model diagram of the effect of obsessive passion.

Bootstrap sampling method was used to test the mediation effect. The path coefficient results were shown in Figures 1, 2 and Table 3. Academic passion and academic self-efficacy significantly mediated the relationship between academic hardiness and subjective well-being, with a total standardized mediation effect value of 0.09 (harmonious passion) and 0.08 (obsessive passion), accounting for 30.27% (harmonious passion) and 25.26% (obsessive passion) of the total effect of academic hardiness on subjective well-being, respectively. Two significant mediating chains were identified in this study. First, the mediating effect 0.05 (harmonious passion) and 0.09 (obsessive passion) generated by Path 2 of “Academic hardiness → Academic self-efficacy → Subjective well-being,” accounting for 16.60% (harmonious passion) and 28.40% (obsessive passion) of the total effect. Second, the mediating effect 0.02 (harmonious passion) and 0.003 (obsessive passion) generated by Path 3 of “Academic hardiness → Academic passion → Academic self-efficacy → Subjective well-being,” accounting for 6.19% (harmonious passion) and 1.02% (obsessive passion) of the total effect. The 95% confidence intervals for the above two paths did not contain zero, indicating that the indirect effects were significant.

**TABLE 3:** Test of standardized bootstrap intermediary effect.

Effect	Path	Effect value	95% LLCI	95% ULCI	Effect proportion
Direct effect	Academic hardiness→Subjective well-being	0.23 (0.24)	0.14 (0.16)	0.31 (0.32)	69.73% (74.74%)
Indirect effect	Mediating effect 1	0.02 (−0.01)	−0.02 (−0.03)	0.06 (0.0005)	7.48% (4.16%)
	Mediating effect 2	0.05 (0.09)	0.01 (0.04)	0.10 (0.15)	16.60% (28.40%)
	Mediating effect 3	0.02 (0.003)	0.003 (0.001)	0.04 (0.007)	6.19% (1.02%)
Total indirect effect		0.09 (0.08)	0.09 (0.02)	0.35 (0.14)	30.27% (25.26%)
Total effect		0.32	0.26	0.39	100%

Mediating effect 1: Academic hardiness → Academic passion → Subjective well-being; Mediating effect 2: Academic hardiness → Academic self-efficacy → Subjective well-being; Mediating effect 3: Academic hardiness → Academic passion → Academic self-efficacy → Subjective well-being; The data in parentheses represents obsessive passion.

## Discussion

The correlation and mediation model analysis of this study revealed a significant positive relationship between academic hardiness and subjective well-being among teenagers, supporting Hypothesis 1. Research indicates that individuals possessing positive intrinsic traits exhibit enhanced physical and mental health when confronted with stress (e.g., Zhang and Wang, 2012). The positive quality of academic hardiness may positively correlate with the subjective well-being of teenagers in several ways. First, individuals with high academic hardiness are more accepting of academic stress. They are more likely to be proactive in increasing their academic engagement when faced with difficult academic tasks. They may be less likely to engage in negative defensive responses, such as withdrawing and abandoning goals. For example, one study found that academic hardiness mitigates the negative effects of stress on college students’ academic achievement (Kamtsios and Karagiannopoulou, 2015). Students with higher levels of academic hardiness also tend to experience less anxiety (Maddi et al., 2011). Second, academic hardiness enables teenagers to confront potential academic failures with increased serenity, avoiding self-denial due to present or future setbacks, thereby preserving a higher level of subjective well-being. The ancient Chinses proverb, “Learning is like rowing a boat against the current,” suggests that learning requires sustained effort, and that academic hardiness is an important quality that helps teenagers to sustain their efforts in the sea of learning.

The findings of this study demonstrated a substantial positive link between academic hardiness and both harmonious passion and obsessive passion, with the correlation between academic hardiness and harmonious passion being more substantial than that with obsessive passion. Second, harmonious passion and obsessive passion had no significant mediating effect between academic hardiness and subjective well-being. The coefficient of the negative association path between obsessive passion and subjective well-being was small, although it reached a statistically significant level. These results cannot support Hypothesis 2. The previous studies found that harmonious passions positively predicted subjective well-being, whereas obsessive passions negatively predicted subjective well-being (Niemic and Ryan, 2009; Ruis-Alfonso and Leon, 2016). The reason why the results of the present study are inconsistent with existing research may be because subjective well-being contains both cognitive and affective dimensions. Academic passion is associated with the affective experience of subjective well-being, and academic passion is a relatively short-lived affective experience. A meta-analysis of 94 studies also found an equivocal relationship between obsessive passion and negative affect (Curran et al., 2015). In some settings, it also may be difficult for individuals to distinguish harmonious passion from obsessive passion. Another possible reason that the relationship between academic passion and subjective well-being is also influenced by other factors (e.g., the influence of academic self-efficacy, which is analyzed subsequently in this paper).

The results of this research demonstrate that academic self-efficacy mediates the connection between academic hardiness and subjective well-being, supporting Hypothesis 3. The positive relationship between individual academic self-efficacy and subjective well-being (Chen et al., 2021; Wang et al., 2017; Lei and Huang, 2015), as well as the positive relationship between academic hardiness and academic self-efficacy (Kuo et al., 2021; Tan et al., 2021; Wong et al., 2019; Cheng et al., 2019) have been reported in existing studies. Hardy students are committed to academic activities, which enhances their confidence in academic achievement and outcomes, resulting in higher levels of academic self-efficacy. Additionally, the chain mediation pathway analysis results in this study indicate that both harmonious passion and obsessive passion are positively correlated with subjective well-being through the mediating variable of academic self-efficacy. Previous studies have found that learning passion is positively correlated with individuals’ learning persistence (Liang and Tian, 2018). Academic harmonious passion represents emotional experiences when individuals actively engage in academic activities and feel a sense of control over the tasks. In this situation, individuals are more likely to receive positive feedback both emotionally and behaviorally, thus promoting the enhancement of academic self-efficacy. Obsessive passion arises when an individual participates in academic activities due to external pressures or expectations, often feeling that academic tasks are beyond their control or not voluntary. While individuals with obsessive passion may increase their academic engagement, leading to opportunities for improved academic performance and enhanced academic self-efficacy, this experience is not entirely positive in terms of emotional outcomes. Consequently, the relationship between obsessive passion and subjective well-being is relatively complex. Although obsessive passion may negatively correlate with subjective well-being due to emotional experiences, the behavioral outcomes contribute to improving academic self-efficacy and positively correlate with subjective well-being, suggesting a mutually offsetting effect between the two. This study identified a masking effect in the relationship between obsessive passion and subjective well-being, which may explain why the coefficients in the correlation analysis between obsessive passion and subjective well-being was not significant, while the coefficient in the mediating path analysis was small. Academic self-efficacy is an individual’s assessment of their academic abilities, this cognition tends to be relatively stable, resulting in a closer correlation with individual subjective well-being. Obsessive passion may stem from an individual’s desire for success or the need to avoid external punishment, leading to the internalization of these pressures or expectations while participating in learning activities. Although individuals with obsessive passion may experience increased academic self-efficacy, they may also feel anxious, stressed, and potentially fatigued. Li et al. (2021) found that college students experiencing academic obsessive passion are more likely to adopt avoidance-oriented problems solving strategies.

**FIGURE 3:**
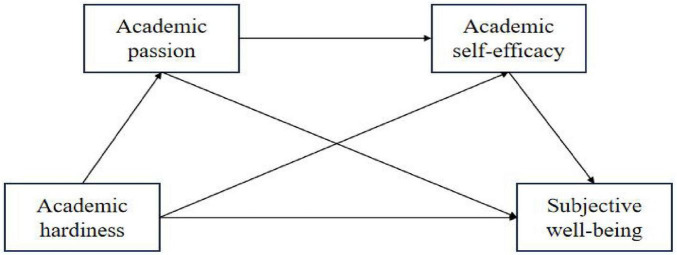
Hypothesized model.

The combination of two chain mediation models (Figures 1, 3) reveals that the proportion of direct effects between academic hardiness and subjective well-being is much greater than the proportion of indirect effects generated by the chain mediation pathway. This indicates that academic hardiness is most closely related to the subjective well-being of teenagers. Tan et al. (2021) found that the commitment of academic hardiness is the most important factor in positively predicting self-efficacy in scientific learning. Secondly, the correlation between academic hardiness and harmonious passion is stronger than that between academic hardiness and obsessive passion. This suggests that teenagers with high levels of academic hardiness are more likely to experience academic harmonious passion, which is more conducive to improving their learning emotion experience. Thirdly, although both academic harmonious passion and obsessive passion positively correlate with academic self-efficacy, the correlation between academic harmonious passion and academic self-efficacy is stronger than that between obsessive passion and academic self-efficacy. This indicates that individuals who experience academic harmonious passion are more likely to have higher academic self-efficacy than those who experience obsessive passion. Larionow and Gabrys’ (2024) also proposed that harmonious passion play a more influential role in various aspects of students’ lives than obsessive passion. The results of this study indicated that implementing school support programs targeted at the development of academic hardiness is of positive significance for improving teenagers’ positive academic emotional experience, academic self-efficacy and subjective well-being.

## Conclusion

Academic hardiness is the variable most closely related to subjective well-being. Academic self-efficacy plays a significant mediating role between academic hardiness and subjective well-being. Academic hardiness is positively associated with subjective well-being through the chain mediation path from academic harmonious passion (or obsessive passion) to academic self-efficacy.

## Limitations and future directions

Some intellectual factors may also influence the relationship path of this study, and subsequent studies can examine these factors comprehensively. Longitudinal studies can explore the causal relationship between variables and overcome the shortcomings of the cross-sectional survey method used in this study. Using experiments that induce harmonious academic passion and obsession academic passion can help research to clarify the complex relationship between academic passion and other variables. Expanding sample variability could improve the generalizability of the findings and overcome the shortcomings of convenient sampling in this study.

## Data Availability

The raw data supporting the conclusions of this article will be made available by the authors, without undue reservation.
